# PD122 Evaluating The Efficacy Of Cytokine Filtration In Cardiac Surgery For Endocarditis: A Comprehensive Study

**DOI:** 10.1017/S026646232400360X

**Published:** 2025-01-07

**Authors:** Marcus Carvalho Borin, Carina Rejane Martins, Daniel Pitchon dos Reis, Geraldo Jose Coelho Ribeiro, Julia Teixeira Tupinambas, Karina de Castro Zocrato, Lelia Maria de Almeida Carvalho, Marcela Pinto de Freitas, Maria da Gloria Cruvinel Horta, Mariana Michel Barbosa, Mariza Cristina Torres Talim, Sergio Adriano Loureiro Bersan, Silvana Marcia Bruschi Kelles

## Abstract

**Introduction:**

Despite medical advancements, endocarditis still results in high mortality rates. Surgery, while often essential, elevates the risk of hyperinflammation, sepsis, and cytokine release. The use of a cytokine filter to prevent this remains controversial. This study reviewed existing literature to assess the efficacy of cytokine filters and to support its integration into supplementary health services.

**Methods:**

An exhaustive search of the MEDLINE, Cochrane Library, Embase, LILACS, and CytoSorbents Corporation databases was conducted to identify relevant meta-analyses and systematic reviews. The study focused on randomized controlled trials and case series studies assessing the efficacy of cytokine filtration. Key variables considered were the duration of antibiotic treatment, severity of endocarditis, and surgical treatment rationale. These factors were crucial for evaluating clinical outcomes and patient survival after surgery.

**Results:**

The systematic reviews yielded mixed outcomes. Two found no benefits for hemoadsorption, while one found that it reduced mortality rates and intensive care unit stays based on observational studies. Randomized controlled trials, however, showed no significant impact for cytokine filters on mortality rates or postoperative hemodynamic parameters. In contrast, case series studies reported potential benefits, but these results were confounded by biases in patient allocation and failure to account for critical variables like antibiotic treatment duration, case severity, and surgical rationale. These discrepancies highlight the complexity of evaluating the effectiveness of cytokine filtration in surgical settings.

**Conclusions:**

Randomized and non-randomized controlled trials on the role of cytokine filters in cardiac surgery for endocarditis reported contradictory findings. Only case series studies suggested benefits from cytokine filters, necessitating further high quality research before recommending their widespread use. Understanding the implications of these results is essential, underscoring the need for more rigorous studies to resolve these inconsistencies.

